# A randomized controlled trial of analogue pharmacogenomic testing feedback for psychotropic medications

**DOI:** 10.1016/j.pecinn.2022.100119

**Published:** 2022-12-16

**Authors:** John Young, Aileen Jimenez, Madeline Pruett, Laken Hancock, McCall Schruff

**Affiliations:** a University of Mississippi, Department of Psychology, 207 Peabody Hall, University, MS 38677, USA; b University of North Carolina at Chapel Hill, School of Pharmacy, 301 Pharmacy Lane, Chapel Hill, NC 27599, USA

**Keywords:** Pharmacogenomic testing, Medical decision-making, Psychotropics, Patient interaction, Clinical feedback

## Abstract

**Objective:**

To examine the impact of various presentations of pharmacogenomic testing results using a published, color-coded decision support tool (DST) format as a standard stimulus to list possible medications.

**Methods:**

Participants were randomly assigned to groups and asked to decide which psychotropic medication they would prefer if depressed. Three of the groups varied the color-coded category of fluoxetine and received a statement indicating that this was the most prescribed drug for depression. A fourth control condition omitted base rate information. Participants also provided detail about their decision-making processes through a qualitative interview.

**Results:**

Comparison of the first three groups indicated that significantly more participants selected medications from the highest category of likely effectiveness when fluoxetine appeared in this list. Comparison of the control group to its relevant analogue suggested no significant differences in selection strategy. Qualitative interview responses indicated participant comfort with genetic testing despite awareness of having very limited understanding of these techniques and their implications.

**Conclusions:**

Both DST color-coding and base rates were influential in driving drug selection decisions, despite most participants indicating they did not understand this information.

**Innovation:**

Efforts to standardize pharmacogenomic stimuli may lead to advances in methods of studying quantifiable healthcare decisions. Attention to the context for presenting test results may also be a useful source of understanding patient responses, particularly regarding complex tests that are likely to be interpreted heuristically.

## Highlights

• Pharmacogenomic decision support tools enable development of standardized methods.
• Using genetic information for healthcare decision-making relies on multiple factors.
• Caution is warranted in conveying pharmacogenomic information to patients.

## 1 Introduction

Pharmacogenomics is the study of how an organism’s entire genetic structure (or genome) influences its response to drugs [1]. The frequency of pharmacogenomic testing as part of more general clinical decision-making is increasing and these tests appear likely to become a ubiquitous part of applied healthcare in the near future [[1], [2], [3], [4], [5]]. For example, there are currently more than 400 FDA drug label advice statements concerning genetic variation [6], and basic education in pharmacogenomic testing is becoming standard for pharmacy training programs [5,[7], [8], [9], [10], [11], [12], [13]]. Despite notable field-wide difficulties with implementation [14] the rapid diffusion of these technologies and potential for strong influence on individual healthcare determination belies the need to understand how patients consider their results and integrate findings into their decision-making processes.

Burgeoning research in this area suggests that many people receiving pharmacogenomic test results do little to coordinate this information with their medical providers [[15], [16], [17], [18], [19], [20]], despite follow-up professional consultation being an expressly indicated desire and/or observed outcome of receiving pharmacogenomic test results [[21], [22], [23]]. Negative emotional reactions to the results of genetic testing (including whole genome testing) have not been notable on average, although a small (but not inappreciable) percentage of participants have exhibited intense responses with lasting ramifications [[24], [25], [26], [27], [28], [29], [30], [31], [32], [33], [34]]. Overall, literature in this domain is formative and the rapidly proliferating integration of these techniques into the broader healthcare environment has contributed to a lag in understanding the relevant human factors. It is particularly problematic to interpret extant studies as a whole given the considerable diversity in testing methods, targets, disease states, and choice of psychosocial variables used as dependent measures.

Fortunately, some published pharmacogenomics decision-support tools (DSTs) exist that may help clarify and standardize research in this area. Myriad Health’s GeneSight™ testing product, for example, has published several accounts of its psychiatric DST that appear in essentially the same form as that distributed commercially [[35], [36], [37], [38], [39], [40]]. This process entails delivery of DSTs to clinicians, who then convey results to patients (and often provide them a copy). When used to guide clinical care, this test has shown promise for increasing depression diagnostic remission and reducing the number of medication changes in previous randomized controlled trials [36,39], indicating potential practical applicability. Using GeneSight’s feedback tool to design research methodologies may thus offer a consistent means of understanding how people orient to the results of complex genetic tests in some contexts related to drug selection. The fact that this particular DST provides guidance for psychotropic prescriptions associated with relatively high base-rate conditions potentially makes it of even greater utility to real decision-making. There is also evidence that individuals with psychiatric difficulties have strong interest in receiving and understanding pharmacogenomic testing to calibrate their psychotropic medication selections [41]. Therefore, the current study constructed a randomized trial examining hypothetical pharmacogenomic testing results using GeneSight’s DST as the template for conveying information, with the primary goal of discerning what features of the DST were most influential in decision-making. A secondary goal of the study included gathering participants’ impressions of possible cognitive, behavioral, and emotional reactions through qualitative interviews.

## 2 Method

### 2.1 Study design

**Fig. 1:**
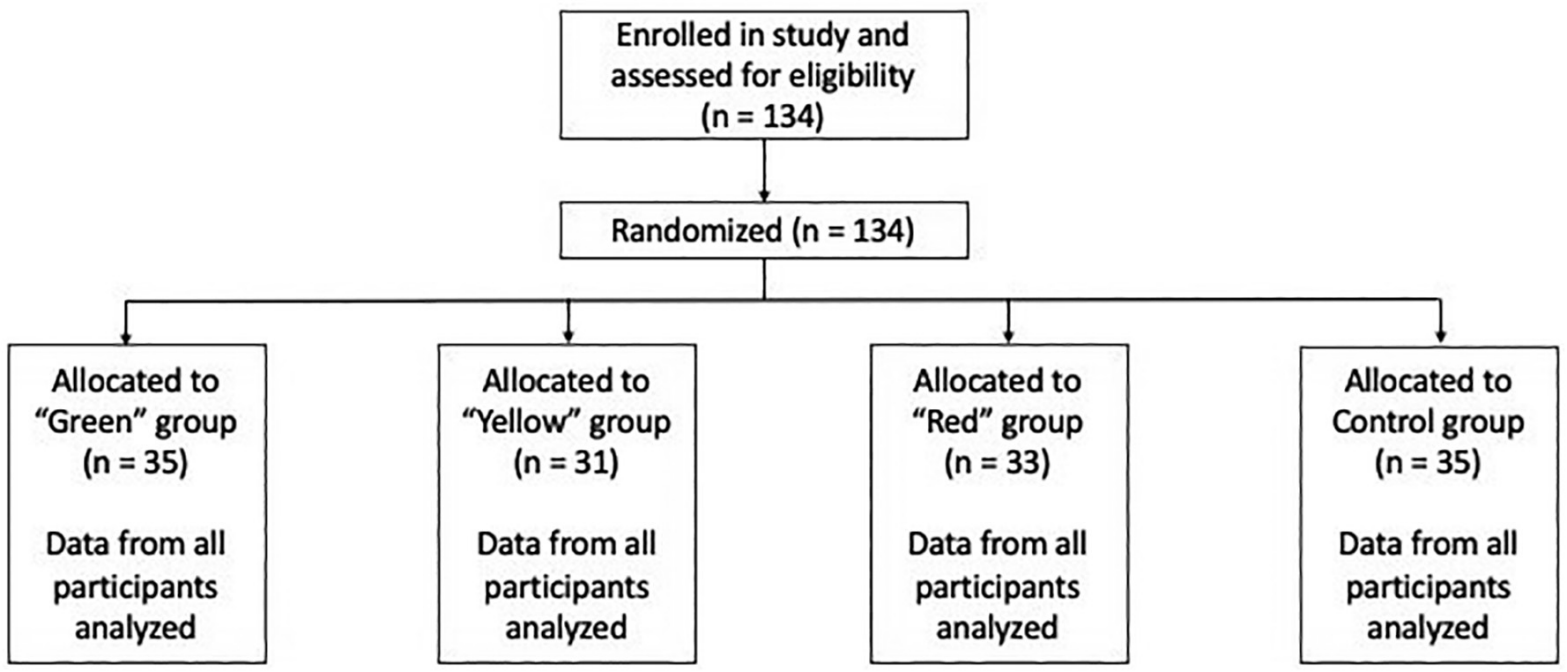
CONSORT diagram.

The study implemented a randomized controlled, mixed methods design with college students recruited from a large University in the southeastern United States. The only inclusionary criterion was being at least 18 years of age (and no exclusionary criteria were implemented). Assignment followed CONSORT guidelines, which are detailed in Figure 1.

### 2.2 Participants

The current study involved analogue examination of 134 undergraduate students ages 18 – 25 (mean age = 19.04; SD = 1.49; 64.2% female). Self-reported ethnicity indicated that the majority of participants identified as White (70.9%), followed by Black (21.6%), Asian (3.0%), Latinx (3.0%), and other (1.5%). All students were compensated with course credit for their participation.

### 2.3 Ethical considerations

The study received ethical review and approval by the Institutional Review Board of the University where the work was conducted. Informed consent was obtained from each participant, who had an opportunity to ask questions and was informed that they could withdraw at any point without negative ramifications. Similarly, all other standards of the Declaration of Helsinki were also upheld across the entire study. All participant data were de-identified when they were recorded, thus no one could be matched to individual responses upon completion of the study.

### 2.4 Measures

The Depression, Anxiety, and Stress Scales, 21-Item Version (DASS-21) is a widely used, public domain measure with substantial psychometric support [[42], [43], [44]]. It has three even-length scales assessing symptoms of depression, anxiety, and stress experienced over the past two weeks. Responses are given on a 4-point Likert-type scale and summed to yield three specific subscale scores, which can be interpreted in comparison to established norms [42]. The Positive and Negative Affect Scales (PANAS) is a well-known measure of positive and negative affect comprising two, 10-item subscales [45]. Respondents use a 5-point Likert-type scale to endorse their degree of experiencing each emotional state within the past week.

Follow-up questions concerning participants’ reactions to pharmacogenomic stimuli were asked using a semi-structured interview constructed for this study (see Appendix A). These primarily qualitative questions were designed to assess respondent orientation to the topic, possible emotional/behavioral reactions, and points of confusion in understanding feedback. Some questions also involved quantitative appraisal in the form of likelihood ratings for various follow-up activities (e.g., taking medication vs. pursuing psychosocial treatment for depression). Additionally, the format of the interview allowed for departure from the structure of questions in order to enable the broadest possible view of respondent reactions.

### 2.5 Procedure

Participants were recruited from psychology classes through online announcements. Upon presentation to the study, they first completed the DASS-21 and PANAS self-report instruments before being randomly assigned to a group. Each condition heard a set of instructions to guide their hypothetical consideration of fictitious pharmacogenomic testing results, which were read aloud by a research assistant and simultaneously displayed in written form on a computer screen. Dashboard summaries for antidepressants, designed to closely emulate those delivered with GeneSight™ testing results, were presented immediately following this description (see Appendix B). These used a color-coding system to group medications into categories that loosely followed the same design as a stoplight. “Green” category drugs were those most likely to be metabolized well for a given person, “yellow” were those that required some caution, and “red” indicated that additional caution and frequent monitoring from a qualified prescriber were recommended.

Dashboards were very similar across groups except for the categorical placement of fluoxetine (which varied between being classified as “green,” “yellow,” or “red”) and the movement of one other medication to ensure that each list was of equivalent length across groups. Fluoxetine was chosen as the target for manipulation given its recognizability and the assumption that more participants would have prior knowledge of the medication in comparison to the others (particularly when referred to by its trade name, as it is on the DST). Additionally, the statement that preceded dashboard presentation varied slightly in a fourth comparison group to avoid any mention of a specific medication or base rates of prescriptions (which was otherwise identical to and used the same color-coded DST as the “yellow” group). The general statement appears below and the text in bold was omitted for the control condition:

I want you to pretend that you are very depressed and went to see a psychiatrist who suggested that you should undergo genetic testing before starting any medication. As the psychiatrist explained, this testing looks at your genetic code and predicts which medications are likely to work best and worst. I’m going to give you a feedback form that shows the results of this genetic testing. It’s pretty similar to how some of these forms actually look, and all the medications listed are real. Even though the results are fake, think of them as though they were the real results of your own genetic testing. **Also, be aware that the most commonly prescribed medication for depression for people in your age group is fluoxetine/Prozac.** Take a minute to look over the form and learn what you can, and then I’ll ask you a few questions about your reactions.

Fluoxetine was in the “yellow” category for all participants assigned to the control group. When compared to the “yellow” group that heard the entire statement this allowed investigation of the relative impact of including information about prescription base rates in addition to the results of pharmacogenomic testing.

After participants reviewed the form, they were asked to select which medication they would prefer (with the indication that they would be taking one of the medications on the list, whether or not that aligned with their individual proclivities). Next, research assistants (the 2^nd^ – 5^th^ authors) asked all participants questions from the semi-structured interview to gain insights into their orientation to and understanding of pharmacogenomic testing. As indicated above, these prompts (generated for the current study) were the starting point for a more general conversation and respondents were also free to provide any tangential information that they wished. Research assistants took notes capturing participant responses, with an emphasis on verbatim transcription of representative quotes. The complete content of the semi-structured, qualitative interview and an example dashboard appear in Appendix A and B (respectively).

### 2.6 Data analysis

Quantitative comparisons of the frequency of drug selection were made between the “green,” “yellow,” and “red” groups using a chi square test of independence assuming equivalent probability of choosing a medication from any one of the categories. A similar procedure comparing the control and “yellow” group was also conducted to examine the impact of the base rate statement on this same decision. Anxiety, depression, stress, positive and negative affect scores, and likelihood estimations from questions 3, 4, and 7 on the semi-structured interview (see Appendix A) were calculated by group to examine potential differences that could have influenced evaluation of the target construct (i.e., medication choice).

Qualitative information provided by interviewees was also coded by the first author into themes on the basis of similarity in responses. This followed the general framework suggested by seminal researchers in qualitative methods (46) in what was later classified as a conventional approach (47). In short, the entire body of participant responses was reviewed to generate some understanding of the overall quality of reactions without connection to theory or preconceived notions. Each response was then re-reviewed and consolidated into categories that summarized meaningful content across respondents. These categories were in turn condensed to reflect general themes, the most frequent of which are described in the results.

## 3 Results

### 3.1 Overview

The results that follow are divided in accord with the goals of the study. First, outcomes of the RCT were examined to understand which features were most salient in guiding participant decisions and any potential moderators of the decision-making process. Second, qualitative responses were coded and summarized to facilitate ideas about potential reactions to receiving this information in a healthcare setting and concerns about genetic testing more generally.

### 3.2 Randomized trial outcomes

Chi-square comparisons across the “green,” “yellow,” and “red” groups yielded a significant difference (Χ^2^ (4) = 23.29; p < 0.001), indicating that the frequency with which drugs from each category were selected varied as a function of group (see Table 1). Participants in the “green” group almost universally chose a medication from the “green” category, with 80.0% of all respondents choosing fluoxetine. In the two instances (5.7%) of “green” respondents choosing a “yellow” category medication both participants independently indicated that their choice was related to their own use of a given drug and perception that it was effective for reducing unspecified psychiatric symptoms. Participants in the “yellow” condition selected yellow category medications at a higher rate (32.2%) than the “green” group, with 90% of those choosing a yellow category medication selecting fluoxetine. The dispersion of categories was more diverse for participants in the “red” group with the majority (72.7%) choosing a green category medication and a substantial minority (21.2%) choosing a red category medication. Of the latter group, 100% selected fluoxetine as their medication of choice. Alternatively, additional comparisons between the “yellow” group and the control condition (which had identical procedures except for the omission of information about base rate in the latter) showed no differences in frequency of selection by category (Χ^2^ (2) = 4.33; p = 0.12). Of note, however, only two (5.7%) of the control group selected fluoxetine specifically.

**Table 1:** Categories of medications selected by group.

Group	Medication category		
	Green	Yellow	Red
Green a	33	2	0
Yellow^b^	20	10	1
Red^c^	24	2	7
Control^d^	29	4	2

a 28 chose fluoxetine.

b 9 chose fluoxetine.

c 7 chose fluoxetine.

d 2 chose fluoxetine.

The overall results of the DASS-21, PANAS, and likelihood questions from the semi-structured qualitative interview can be seen in Table 2. This entailed group-level scores similar to normative values for the standardized instruments, indicating that the participants did not exhibit overt signs of psychopathology (on average). Mean comparisons (ANOVAs) of scores across groups yielded no significant differences on any subscale or likelihood rating, suggesting relative equivalence in terms of factors that might bias perceptions of pharmacogenomic results.

**Table 2:** Self-report scores by group.

	Green (n = 35)	Yellow (n = 31)	Red (n = 33)	Control (n = 35)
Depression	2.73 (3.54)	2.33 (2.35)	2.58 (3.43)	2.70 (3.66)
Anxiety	3.68 (2.86)	2.71 (2.31)	2.91 (3.21)	2.32 (2.31)
Stress	5.41 (4.25)	5.57 (4.03)	5.79 (3.99)	3.85 (2.88)
Positive Affect	39.00 (5.40)	36.43 (7.36)	38.70 (5.07)	39.29 (3.86)
Negative Affect	21.97 (6.77)	21.14 (6.70)	21.28 (6.19)	19.57 (5.21)
Medication likelihood	73.51 (27.32)	67.13 (26.12)	69.48 (26.44)	73.14 (26.64)
Therapy likelihood	82.29 (21.12)	84.19 (20.09)	87.88 (21.33)	86.86 (18.79)
Genetic testing likelihood	89.86 (16.16)	81.87 (22.88)	86.06 (21.71)	84.43 (21.72)

### 3.3 Participant feedback on DST

Common themes that emerged from qualitative responses included 1) reporting that the color of the category dictated medication decision (e.g., “I wanted to stay in the safe green box and then chose one because I liked the name.”); 2) a desire to do additional, independent research about medication effects (e.g., “I’d want to research the green drugs, maybe ask my family if anybody had taken one of them.”); 3) indication that the results of testing were conveyed clearly in the DST (e.g., “It was really clear which ones they said would work.”); 4) a suggestion to include known side effects associated with each drug listed (e.g., “It would be better if they told you what the side effects of each medicine was.”); 5) little understanding of the process of or scientific support for genetic testing (e.g., “I don’t know what I don’t know about this stuff.”); and 6) limited concern for negative impact of genetic tests, despite indication of knowledge deficits (e.g., “I don’t know enough to be concerned”). Among the few who did express reservations, the most frequent theme was concern about how their genetic testing results might get used, particularly in terms of the potential for this information to be delivered to insurers or governmental agencies. A smaller number of individuals reported lack of certainty about the validity of these testing methods and indicated concern that over-reliance on their use could lead to inaccurate decision-making by providers. Finally, in a single instance that was notable for its deviation from scientific feasibility, one respondent articulated fears that genetic material collected during testing could be used to clone people without their consent (i.e., “I heard that people could get cloned if they get your DNA and I wouldn’t want that happening if I didn’t know about it.”).

Information gathered in response to question 8 indicated that most respondents would be upset by results indicating that all of the medications were in the “red” category. This response was given by nearly every respondent, irrespective of the individual’s stated likelihood of seeking medication as an intervention for depression. Many interpreted this as a sign that their depression was extremely severe and/or would never improve, despite no indication that the test conveyed anything about specific symptom expression or severity. In addition to generally describing their emotional states in terms of dejection, hopelessness, and disappointment, many participants also conveyed that they would be angry that medications were offered as a potential treatment option when test results indicated that this would not be effective.

## 4 Discussion and conclusions

### 4.1 Discussion

The main results of the study indicated that individual choices were influenced by the context in which information was presented, particularly the organizational format of the DST. This appeared to interact with information about base rates such that alignment between these two sources of information (i.e., the “green” category) facilitated extreme consistency in selection of fluoxetine as the preferred medication. It appeared that most deviations from selecting “green” medications were in favor of fluoxetine in the “yellow” and “red” groups, with 100% of the latter choosing fluoxetine. This suggests that information about base rates was more influential to the degree it diverged from indications given in the DST. Regardless, it was also notable that these deviations represented only a minority of cases, with 20.9% of respondents choosing anything other than a “green” category drug. The effect of conveying base rate information could also be seen in comparing the “yellow” and control groups, wherein the patterns of categorical endorsement were similar (i.e., mostly “green” selections) but the specific selection of fluoxetine was not (with fluoxetine rarely being selected in the control group). Without additional information to guide decisions, the control group effectively appeared to default to a “green” medication and exhibited some unexplained variability outside of that category. Although the participants choosing a medication from a different category did not independently offer a rationale that could elucidate their reasoning, it could be postulated that their selection of “yellow” or “red” category drugs was attributable to previous experience (similar to what was stated by the two “green” respondents selecting “yellow” medications). Taken together, it appeared that information about base rates of prescription was potentially meaningful to respondents, but was nonetheless secondary to their examination of the DST and inference that “green” drugs were the best selections.

It is important to note that this interpretation is somewhat more nuanced than might be expected, particularly if respondents were generally not familiar with most of the medications listed. Given that individual exposure to information about a specific drug is likely to be highly variable in the population at large, this complicates communication strategies that may facilitate optimal use of pharmacogenomic results. This problem is further compounded when considering medications that are less widely used or recognized (e.g., cancer drugs), making the potential structure of patient feedback a critical issue in the applied use of these tests. Indeed, previous studies have indicated similarly complex predictors of perceived utility [16,41] and behavioral impacts of screening [24,25,28,29,30,32,33], with no clear emergence of specific, generalizable strategies for conveying and making use of test results.

The need for caution in constructing these methods of patient communication was also supported by qualitative responses, which generally indicated that respondents believed themselves to have an immediate and firm grasp of the complicated information presented. Very few participants expressed concerns about the scientific validity of these tests, and the vast majority seemed to interpret outcomes depicted in the DST as categorical and absolute (i.e., “green” = yes; “yellow” = maybe; “red” = no). Additionally, when asked to explain their reactions if all medications were categorized as “red” most individuals indicated that they would be upset by this outcome and proceeded to offer a variety of incorrect interpretations of the DST to support their reasoning. For example, many participants took this to mean that their symptoms were extremely severe and their hope for improvement was minimal (neither of which was stated or implied by the study materials). This confusion and limited understanding of the actual functions of pharmacogenomic testing are potentially informative for future research in understanding individual orientation to these forms of decision support. Likewise, if similar confusion is found through more empirical, objective studies, this lack of knowledge may suggest the need for broader educational campaigns regarding the actual nature of these tests (particularly their probabilistic rather than absolute nature and the use of ranges rather than coarse categories to describe likely outcomes).

Alterations to the DSTs to reflect these probabilities could be instrumental in this process, particularly for clinicians with some grasp of the multifaceted influences of genotype on phenotype. Similarly, reminders of these other potential influences on medication response could also be useful in promoting accurate decisions. For example, categories could be presented with approximate ranges of effectiveness and reminders that these calculations only consider variability based directly on genetic information (e.g., “Use as Directed” followed by “60 – 70% likelihood of effectiveness based on genetic structure *only*”). Such a presentation would not substantially elongate the information contained in the DST and could engender a much different reaction than the use of distinct categories with no additional context. Likewise, it may be beneficial to remove the color-coding aspect of the DST entirely. Following the convention of a typical traffic light likely reduces the complexity of DST interpretation, but the degree to which that is positive in facilitating accurate decision-making is currently unknown. It could be the case that these decisions can be approached more effectively by deliberately *avoiding* this form of simplification in favor of one that purposefully stimulates more complex consideration (although this is only a hypothetical example in the absence of substantial future study).

Study limitations include the small sample size, all of whom were tested under analogue conditions, and the age group of participants. The latter has the potential to introduce barriers to generalization of results, given that the very young adults participating in the study may have had less knowledge of or contact with the healthcare system than older age individuals. Repeating the study in an applied environment where test results are meaningful to actual healthcare decisions with a wider age range could be beneficial to determining additional factors influencing interpretation. Additionally, the study attempted to use a known DST in standardized way that could facilitate additional research, which restricted the possible methods of providing feedback. Future studies could manipulate the format of information for further comparisons, including through the presentation of likelihood ranges, changing the number of categories presented, rank-ordering medications from most to least likely to be effective, and inclusion of additional information about use of the medications (including side effects, which was mentioned by numerous participants). Similarly, efforts to provide information about the tests themselves, potentially in the form of brief training exercises, could be a worthwhile area of study. Finally, efforts to examine similar issues in healthcare providers may be particularly germane to understanding implementation of these tools in applied practice. Given the extant evidence that few providers have training in the use of pharmacogenomic tests [14,[46], [47], [48], [47], [48], [49], [50], [51]], it is likely that they are also relying on DSTs and feedback from the commercial labs that produce these tests to make decisions (which in turn may influence the decisions of their patients).

### 4.2 Innovation

The current mixed methods study was innovative in at least two respects. The first was prompting development of standardized stimuli for future studies of patient reactions to pharmacogenomic testing. The DST utilized in the current study was closely modeled on one of the more prevalent commercial tools for conveying this information in actual healthcare settings, which could facilitate not only additional research but more rapid dissemination to clinical environments that are already using similar tools. In turn, this could stimulate additional research about optimal methods of discussion of these concepts in applied environments, including revision of DSTs to converge on a standard format in the field. Genetic testing to guide medical decision-making is not yet common but will likely be so across most areas of healthcare in the future. The use of innovations such as DSTs is in its infancy and has not been subjected to sufficient study to enable insights as to the most effective, efficient forms of communication. As such, adapting DSTs to be continually innovative in their reduction of complex information to ingestible forms is crucial to building the infrastructure for tomorrow’s healthcare practice.

Additionally, the incorporation of directing participant attention to base rates of prescribing represents an integration of behavioral economics to this form of decision-making [[52], [53], [54], [55]]. Although this area has been well explored in some aspects of health behaviors, these are typically conceptualized more in terms of specific decisions (e.g., whether to receive a vaccination or not) and less as processes of orienting toward information, attempting to understand, and forming insights that guide decisions more generally. Gathering qualitative feedback about individual thought processes associated with decision-making enabled some formulation of how individuals understood pharmacogenomic tests. These insights could be extended if future work continues to develop standardized methods of presentation and a convention to request informal feedback from participants. Likewise, learning what features of messaging or information presentation “nudge” individuals in particular directions may be beneficial to positively influencing both their specific decisions and broader conceptual understanding of the implications of pharmacogenomic testing.

### 4.3 Conclusion

This study provided indication that participants, all of whom were potential patients who may one day encounter pharmacogenomic testing as a part of healthcare services, were influenced in their decision-making by the means in which information was presented. Visual, categorical organization appeared to play the strongest role in impacting decisions, although its influence was relatively weaker when divergent from information about base rates. The outcomes of individual decisions were also observed in the context of most participants’ full cognizance (which was communicated independently, without solicitation) that they did not understand these tests, the basis for drug categorization, or the distal implications of genomic analysis. This did not limit their assertion that they understood the implications of the DST, however, or their comfort with receiving genetic testing and having the results impact their healthcare services (both of which were generally very strong). Taken together, it is feasible that participants were reducing complex decisions that they had no basis to understand to simple decisions using heuristics [52], which has been demonstrated to have negative implications for rational, appropriate decision-making across contexts [[53], [54], [55], [56], [57], [58], [59], [60]].

## Declaration of Competing Interest

The authors declare no conflicting interests.

## Figures and Tables

**Table:** 

Use as Directed	Use with Caution	Use with Increased Caution and with More Frequent Monitoring
Amitrypline (Elavil)	Clomipramine	Doxepin (Sinequan)
Bupropion	(Anafranil)	Duloxetine (Cymbalta)
(Wellbutrin)	Desipramine	Imipramine (Tofranil)
Citalopram (Celexa)	(Norpramin)	Paroxetine (Paxil)
Mirtazapine	Desvenlafaxine (Pristiq)	Selegiline (Emsam)
(Remeron)	Escitalopram (Lexapro)	Venlafaxine (Effexor)
Sertraline (Zoloft)	Fluoxetine (Prozac)⁎	
Trazodone (Desyrel)	Fluvoxamine (Luvox)	
Vilazodone (Viibryd)	Nortriptyline (Pamelor)	

⁎ Note that the placement of this medication changed depending upon random group assignment (i.e., this was the defining characteristic to distinguish “green,” “yellow,” and “red” groups). This is the default example for the control group and “yellow” group. “Green” and “red” groups replaced fluoxetine with another medication taken from the relevant category in order to keep the lengths approximately equal.

## Appendix A Semi-structured follow-up interview

1) What information did you use to make your decision about the specific drug you’d prefer? Tell me about your thought process and reasoning. If you had all the time you wanted, what additional steps (if any) would you have taken before deciding?

2) How much sense were you able to make of the feedback form? What could have made it easier for you to understand? What other information would you like to have included beyond the list of medications?

3) From 0 – 100%, how likely would you be to seek medication if you were clinically depressed? What else might you do to address your symptoms? Would you have any difficulty with or objections to staying on antidepressant medication for the rest of your life? Why or why not?

4) From 0 – 100%, how likely would you be to seek therapy if you were clinically depressed? What factors would influence your decision to do so (or not do so)? Would you want to take medication at the same time? Between medication and therapy, do you view one of these approaches as superior? Why or why not?

5) Would your decision have been different if you didn’t have the results of genetic testing but were presented with the same list of drugs? How so? What information would you use to make your decision in that case?

6) If this were a realistic situation, what else would you want to know? What kinds of emotional reactions would you likely have? What kinds of additional tests or interactions with healthcare providers would you want?

7) From 0 – 100%, how willing would you be to engage in genetic testing if your doctor recommended it? Does your opinion change in terms of what’s being tested (for example, depression medication vs. likelihood of developing a terminal disease vs. learning about the characteristics you might pass on to your children)? What concerns do you have with the process of genetic testing in general? More specifically and hypothetically, what concerns would you have with using your own results in making important healthcare decisions? If there’s anything that “feels” weird to you about this kind of testing I’d like to hear about that too.

8) Imagine the same situation we just talked through, but every single medication on your feedback form is in the “red” box (meaning they’re all predicted to not work very well for you due to your genetic structure). How would that information affect you? In particular, what kinds of emotional reactions might you have? What would you do to address your symptoms as a result of this information?

9) What else comes to mind that would help us understand people’s reactions to this type of information and/or use of it in making healthcare decisions?
